# A siamese network-based approach for vehicle pose estimation

**DOI:** 10.3389/fbioe.2022.948726

**Published:** 2022-09-02

**Authors:** Haoyi Zhao, Bo Tao, Licheng Huang, Baojia Chen

**Affiliations:** ^1^ Key Laboratory of Metallurgical Equipment and Control Technology, Ministry of Education, Wuhan University of Science and Technology, Wuhan, China; ^2^ Wisdri Utility Tunnel Designing Institute, Wuhan, China; ^3^ Hubei Key Laboratory of Mechanical Transmission and Manufacturing Engineering, Wuhan University of Science and Technology, Wuhan, China; ^4^ Precision Manufacturing Institute, Wuhan University of Science and Technology, Wuhan, China; ^5^ Research Center for Biomimetic Robot and Intelligent Measurement and Control, Wuhan University of Science and Technology, Wuhan, China; ^6^ Hubei Key Laboratory of Hydroelectric Machinery Design & Maintenance, China Three Gorges University, Yichang, China

**Keywords:** siamese network, contrast learning, correlation matrix, pose estimation, feature pyramid network

## Abstract

We propose a deep learning-based vehicle pose estimation method based on a monocular camera called FPN PoseEstimateNet. The FPN PoseEstimateNet consists of a feature extractor and a pose calculate network. The feature extractor is based on Siamese network and a feature pyramid network (FPN) is adopted to deal with feature scales. Through the feature extractor, a correlation matrix between the input images is obtained for feature matching. With the time interval as the label, the feature extractor can be trained independently of the pose calculate network. On the basis of the correlation matrix and the standard matrix, the vehicle pose changes can be predicted by the pose calculate network. Results show that the network runs at a speed of 6 FPS, and the parameter size is 101.6 M. In different sequences, the angle error is within 8.26° and the maximum translation error is within 31.55 m.

## 1 Introduction

The pose of an object is a critical indicator of the state of the object. For dynamic objects, their poses are constantly changing and it is more difficult to estimate than static objects (Ding et al., 2018; He et al., 2020; Tao et al., 2021). For vehicles, the process of changing pose represents the interaction between the vehicle and the environment, which is very important for the vehicle to perceive the environment. Intelligent algorithms have been applied to various fields, and have made a series of amazing achievements (Chen et al., 2021a; Chen et al. 2021b; Chen et al. 2022a; Li et al., 2022; Sun et al., 2022), especially deep learning (Hao et al., 2021; Huang et al., 2022; Sun et al., 2020; Jiang et al., 2021; Yun et al., 2022; Zhao et al., 2022). However, there are still many issues, such as high cost of obtaining and labeling high quality data, which limits the potential of supervised learning (Sünderhauf et al., 2018; Chen et al., 2022b; Chen et al., 2022c). Some deep learning methods are completely data-driven, abandoning traditional frameworks and lacking of analyticity to analyze the results effectively. And the pose change is a relative change, it often requires multiple images in the calculation, which further increases the burden of hardware (Tao et al., 2022a; Tao et al., 2022b).

We propose a Siamese network (Yu et al., 2019; Chicco et el., 2021) based vehicle pose estimation network called FPN PoseEstimateNet, which uses two images as input and constructs a feature extractor by combining the Siamese network with the correlation module. A correlation matrix is used for pose calculation. Instead of using an end-to-end neural network, this paper relies on the traditional framework, uses a neural network instead of the feature extraction component, and uses contrast loss and regression loss constraints to train the network.

The FPN PoseEstimateNet is a high speed and lightweight network, and its pose estimation accuracy is comparable to that of most networks. In addition, this network can decouple the pose estimation process into feature extraction for matching and pose calculation, thus enhancing the interpretation ability of vehicle pose estimation. The main contributions of this paper are as follows.

1) We proposed a Siamese network based feature extraction matching method.
2) We proposed a lightweight vehicle pose estimation network.

## 2 Related work

In recent years, deep learning has developed rapidly in the field of computer vision, and the convolutional neural network (CNN) represented by AlexNet (Krizhevsky et al., 2012) is an early representative. As the research progresses, more and more neural networks with excellent performance have been proposed, such as VGG (Sengupta et al., 2019; Mateen et al., 2019; Tammina et al., 2019), ResNet (He et al., 2016; Targ et al., 2016; Theckedath et al., 2020) and Inception (Szegedy et al., 2015; Szegedy et al., 2016; Szegedy et al., 2017; Ioffe et al., 2015). The commonly used deep learning pose estimation algorithms can be divided into two categories: supervised learning and unsupervised learning.

In supervised learning, Konda et al. (2015) considers the pose estimation as a classification problem. Costante et al. (2016) uses CNN for feature extraction and then for pose estimation. DeepVO (Wang et al., 2017; Lee et al., 2021; Wang et al., 2022) is an end-to-end pose estimation network, which uses a deep recurrent convolutional neural network (RCNN) to input a sequence of images and output the corresponding pose directly, without relying on any module in the traditional pose estimation frameworks. On the other hand, it implicitly models the time and data association models through recurrent neural network (RNN). On the basis of DeepVO, many improvements have been made. Some scholars have integrated curriculum learning and geometric constrains (Saputra et al., 2019a). Saputra et al. (2019b) introduces the mechanism of memory model for enhancing the feature extraction.

In supervised learning, it becomes more and more difficult to label all data. Compared with supervised learning, unsupervised learning has the advantage of using more data and better generalization performance in unfamiliar scenes. The SFMLearner (Klodt et al., 2018; Li et al., 2018; Zhang et al., 2020; Liu et al., 2021; An et al., 2022; Shao et al., 2022) algorithm is a typical unsupervised learning method, which consists of single view depth estimation and multi-view pose estimation. It uses synthetic view as the supervised information for depth and pose estimation. Then, the pixels in the source image are projected to the target image, and the pixel differences are found for the corresponding pixel. However, SfmLearner still has the problems of scale uncertainty and inability to adapt to the moving objects in the scene. Li et al. (2018) proposed to solve the scale uncertainty problem by using the acquired image pairs of binocular camera for learning and the monocular camera for pose estimation. Bian et al. (2019) tried to solve the scale problem by re-projecting the input image into three-dimensional (3D) space to determine the scale and then perform pose estimation. For the problem of scene transformation, GeoNet (Yin et al., 2018) solves the motion problem in static scenes by treating static scenes and object motion as different tasks and learning independently. Ganvo (Almalioglu et al., 2019) uses GAN (Generative Adversarial Network) to generate depth maps directly and uses a temporal recurrent network for pose estimation. Li et al. (2019) directly use the generator in GAN to generate a more realistic depth map and pose.

## 3 Methods

**FIGURE 1:**
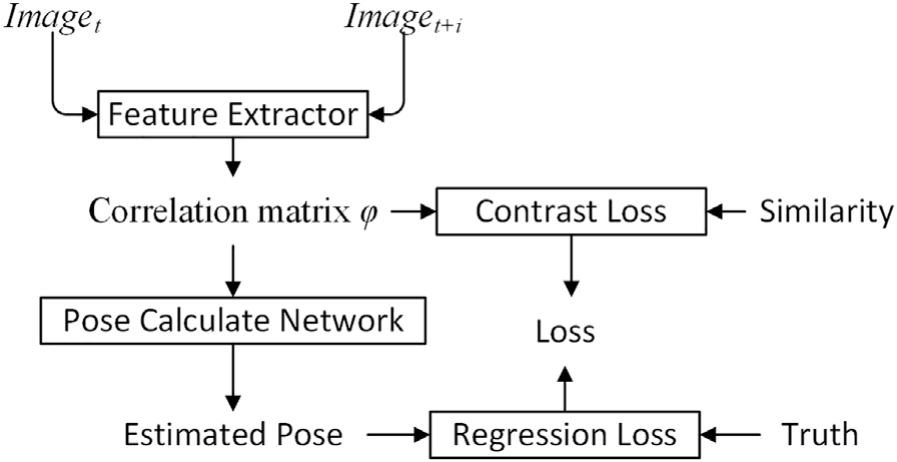
Overview of FPN PoseEstimateNet.

The FPN PoseEstimateNet algorithm is shown in Figure 1. Two adjacent images are processed through the feature extractor to obtain a correlation matrix *φ*. The correlation matrix *φ* is then used in the pose calculate network to predict vehicle pose.

### 3.1 Feature extraction

**FIGURE 2:**
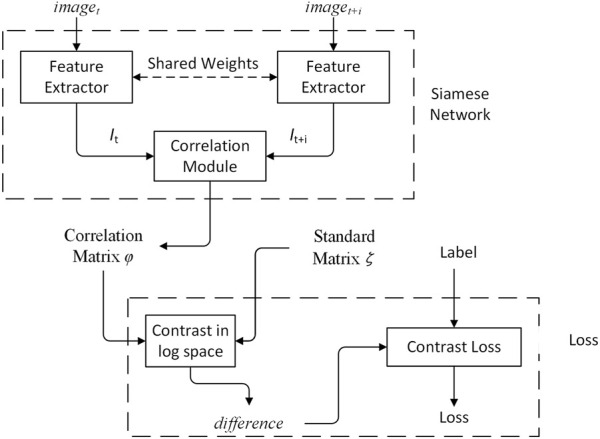
Overview of feature extraction.

The feature extractor adopts Siamese network, shown in Figure 2. The Siamese network is a multiple input single output network. Each input has a corresponding feature extractor, and all feature extractors share the same weights. The structure of the Siamese network ensures that the inputs are mapped to the same feature space. The output is the correlation matrix *φ*, and is constrained by the standard matrix *ζ* and constrative loss.

**FIGURE 3:**
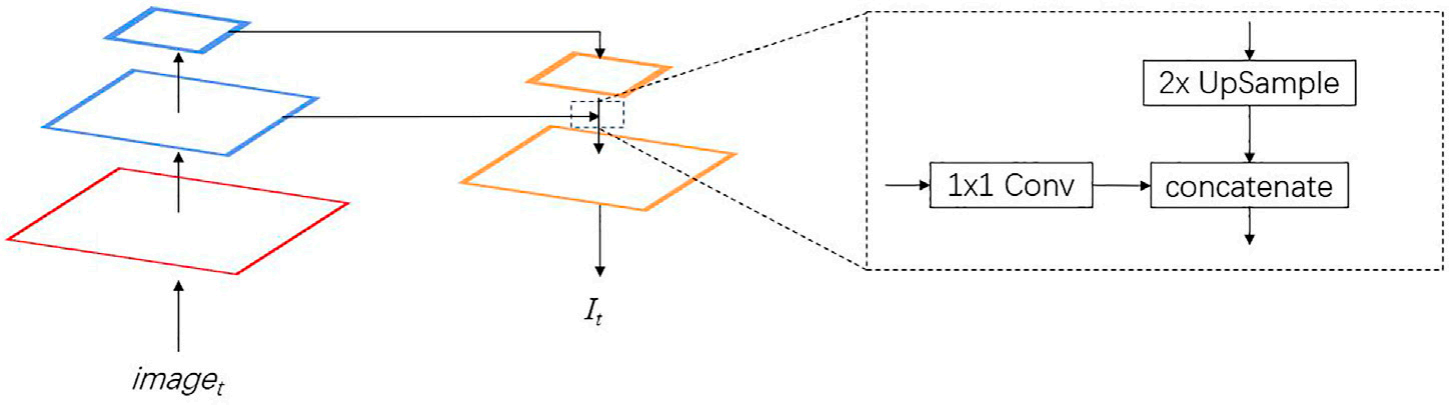
Feature pyramid network for multi scale features fusion.

In feature extraction, multi-scale features are fused by using a FPN. The architecture of the feature extractor is shown in Table 1. Figure 3 shows the multi-scale features fusion. The red part shows the base features. The blue part is the features at different scales. The orange part is the features in the fusion process. For the features at different scales, 1 × 1 convolution kernel is applied to reduce the channel dimension. Multi-scale feature fusion is applied by using upsampling and concatenation as the input to the correlation module. In feature extraction, the small-scale features can retain more underlying basic information and extract more detailed information, while the large-scale features can better represent semantic information. The concatenation of features can preserve the detailed and semantic information.

**TABLE 1:** Architecture of feature extractor of the FPN PoseEstimateNet.

Feature extraction
Input [128,380,3]
Conv [7,7,64,] ReLU stride 2 BN
Conv [55,128] ReLU stride 1 BN
Conv [55,256] ReLU stride 2 BN
Conv [33,512] ReLU stride 2 BN
Conv [33,512] ReLU stride 1 BN
Conv [33,512] ReLU stride 2 BN
Conv [33,512] ReLU stride 1 BN
ZerosPadding [2,1]
Conv [3,31024] ReLU stride 2 BN
ZerosPadding [1,1]
Conv [33,512] ReLU stride 1 BN
ZerosPadding [1,1]
Conv [33,512] ReLU stride 2 BN
ZerosPadding [1,1]
Conv [33,512] ReLU stride 1 BN
ZerosPadding
Conv [11,256] ReLU stride 1
Concatenate
MaxPool [2,2]

### 3.2 Correlation matrix and standard matrix

The patches in the features are encoded into a one-dimensional (1D) feature vector according to Eq. 1. In Eq. 1, *w* represents the width of the features, *x*, *y* represents the coordinates of patches in the features, and *z* represents the patch position in new coordinates. Therefore, *z* represents the spatial information of the patches in the features.

z=x+(y×w) (1)

**FIGURE 4:**
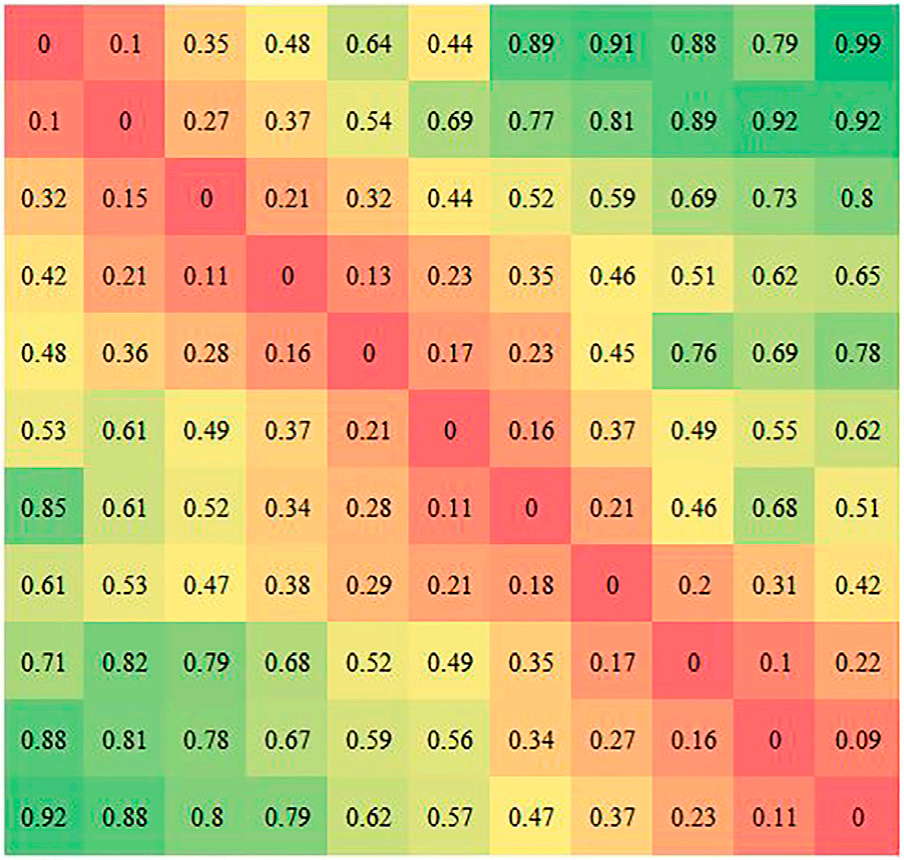
Correlation matrix *φ.*

The input image pairs *image*_*t*_ and *image*_*t+i*_ pass through the feature extractor to obtain a set of feature vectors, ***I***_***t***_ and ***I***_***t+i***_. The correlation module processes ***I***_***t***_ and ***I***_***t+i***_ to obtain a correlation matrix *φ*. The correlation matrix *φ(x,y)* denotes the correlation between the *x*-th patch in ***I***_***t***_ and the *y*-th patch in ***I***_***t+i***_, shown in Figure 4.

We use the distance of the corresponding patches in the high-dimensional feature space as the correlation criteria. The smaller the distance is, the higher the correlation of the corresponding patches is. The smaller the difference is, the greater the similarity is. The distance between corresponding patches can be calculated by Eq. 2.

$$distance\left(I_{t}\left(x\right),I_{t+i}\left(\text{y}\right)\right)=I_{t}\left(x\right)-I_{t+i}\left(\text{y}\right)$$ (2)

Because ***I***_***t***_ and ***I***_***t+i***_ are in high-dimensional feature space, there will be a large bias in using the Euclidean distance. It is necessary to make non-linear transformation of the Euclidean distance. Moreover, the Euclidean distance is in (−∞, + ∞), and the range of the interval is too large, which easily leads to instability in the training. The Gaussian function is used to limit the distance in (0, 1], shown in Eq. 3, where *x* denotes the distance between the corresponding patches, i.e. *distance* (***I***_***t***_ *(x),* ***I***_***t+i***_ *(y)*), and *σ* denotes the variance. Gaussian function can reduce the sample variance, which meets the requirement that the stronger the correlation is, the larger the value is. And the *φ* is normalized by rows.

$$\text{Gaussian}\left(x\right)=\text{exp}\left(-{\left(\frac{\text{x}}{2\text{σ}}\right)}^{2}\right)$$ (3)

**FIGURE 5:**
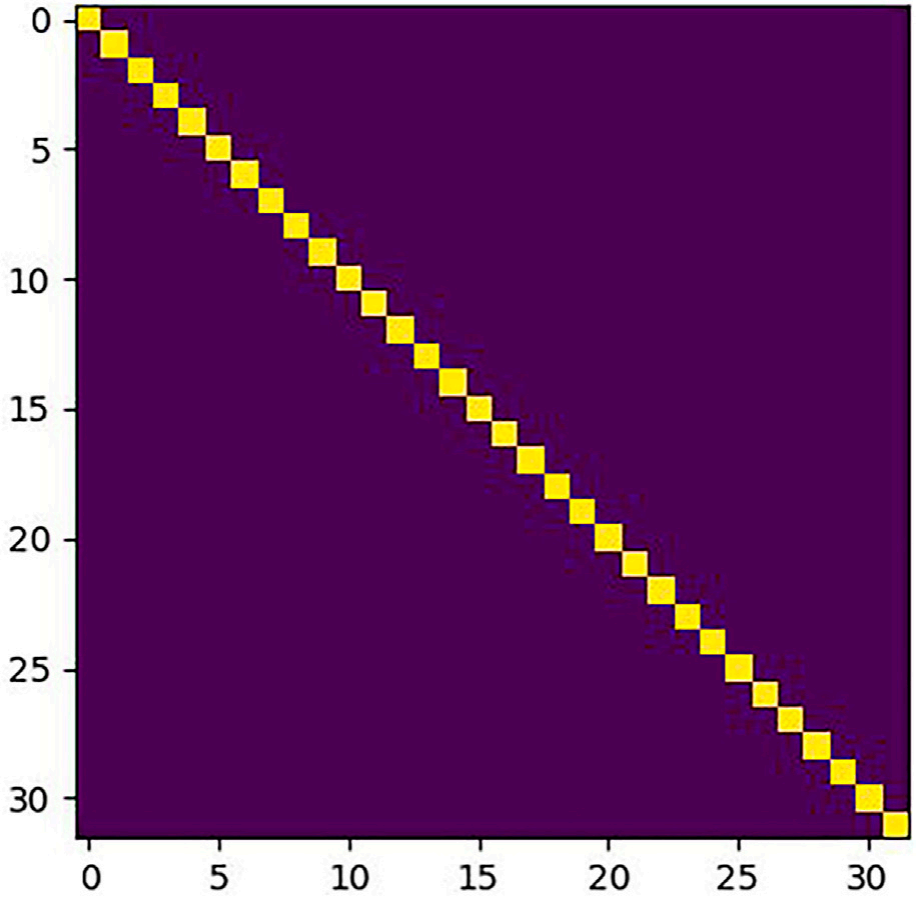
Standard matrix *ζ.*

The correlation matrix *φ* can be obtained by using the correlation module. However, the variation of the weight in the Siamese network has a large impact on *φ*. A standard matrix *ζ* is used to evaluate the variation of *φ.* The standard marix *ζ* is defined as the correlation matrix of the same inputs. Under ideal conditions, the distance between identical patches is zero, and the distance is infinite for non-identical patches. The standard matrix *ζ* is shown in Figure 5. The *X* and *Y* axes represent the positions of the patches in ***I***_***t+i***_ and ***I***_***t***_, respectively.

By comparing the difference between the *φ* and the *ζ* in log space, a comparison *difference* is generated. By using the similarity label and *difference,* the feature extractor can better identify the differences in patches. Thus, the pose changes between *image*_*t*_ and *image*_*t+i*_ can be described by the *difference,* which is the result between *φ and ζ.* The logarithmic distance is used to measure the correlation *φ* and *ζ*, shown in Eq. 4.

difference=log(ζ/φ) (4)

The weighted mean of *difference* is used as the distance to define the pose changes, as shown in Eq. 5, where *difference*_*ii*_ denotes elements on the diagonal and difference_*ij*_ denotes the other elements. Since *ζ* is a constant matrix, the *difference* represents the change in *φ* at the corresponding position. *λ*_*ij*_ is the corresponding weight factor, expressed as the sum of the relative two-dimensional coordinate offset of the *i*-th element of ***I***_***t***_ and the *j*-th element of ***I***_***t+i***_, in the features. And 1 is added to all offset to prevent learning from occurring if the offset is 0.

$$distance=\frac{1}{k^{2}}\sum_{i=1}^{k}\lambda_{ii}difference_{ii}+\sum_{i=1}^{k}\sum_{j\neq i}^{k}\lambda_{ij}difference_{ij}$$ (5)

**FIGURE 6:**
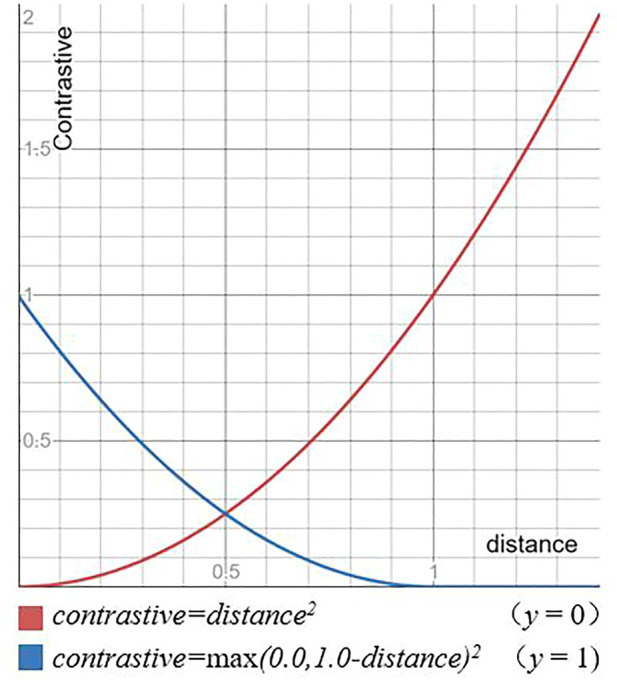
Contrast loss function.

For the *distance*, a contrast loss is used for training. The contrast loss is shown in Figure 6. For positive samples, as shown by the red line, the loss value increases as distance increases. For negative samples, as shown by the blue line, the loss value decreases as *distance* increases until it reaches *margin*.

The contrast loss is shown in Eq. 6, where, *y* is the label, the only values are 0 and 1. And 0 means that the input image pairs are identical, 1 means that they are different, *margin* represents the boundary, and *margin* is taken as 1. The positive label means that the difference is small and the value of *distance* is decreased*.* The negative label means that the difference is large and the value of *distance* is increased. Therefore, the value of *distance* in the case of a label of 1 also reflects the degree of difference, the greater the degree of difference the greater the value of *distance*, the smaller the degree of difference the smaller the value of distance, until it reaches the difference boundary *margin,* when the *distance* exceeds *margin* no longer increases, preventing overfit. The presence of the comparison function allows the network to represent the change of pose.

$$Contrastive=y\times max{\left(0,margin-distance\right)}^{2}+\left(1-y\right)\times distance^{2}$$ (6)

### 3.3 Pose calculate network

**FIGURE 7:**
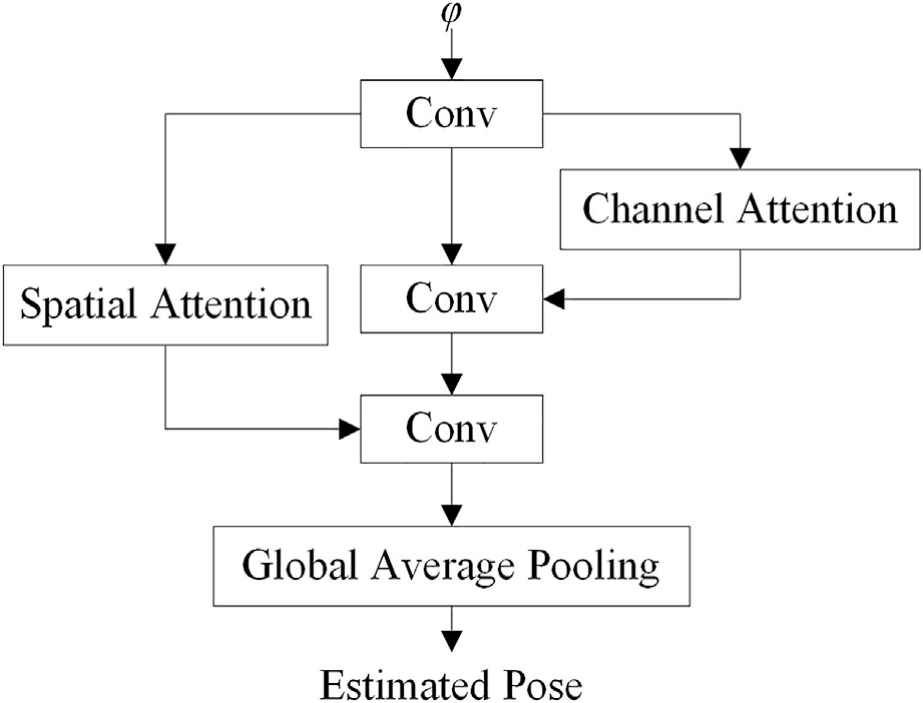
Pose calculate network. Conv for convolution module.

The correlation matrix *φ* represents the matching relationship of the input images. A pose calculate network is designed to process *φ* and recover the poses from *φ*. The basic structure of the pose calculate network is shown in Figure 7.

As the *φ* can reflect the translation and rotation of the vehicle, the pose calculate network uses multiple convolution layers to extract features from the structure. In the process of pose estimation, the channel attention module and the spatial attention module are used to improve the training speed and accuracy.

The network structure of the pose calculate network is shown in Table 2. In Table 2, ChannelAttention1 and ChannelAttention2 are convolution layers in the channel attention network, SpatialAttention1 and SpatialAttention2 are convolution layers in the spatial attention network, and the channel attention network are connected to the backbone network through parallel connection, and the parameter size of the pose calculate network is 5.9 M.

**TABLE 2:** Pose calculate network of FPN PoseEstimateNet.

Pose calculate network
Input [32,32,1]
Conv [3,3,512] ReLU stride 2
Conv [3,3,256] ReLU stride 2
ChannelAttention1 [1,1,32] ReLU
ChannelAttention2 [1,1,256] Sigmoid
SpatialAttention1 [33 128] ReLU stride2
SpatialAttention2 [3,3,1] Sigmoid
Conv [33,128] ReLU
Conv [1,1,3]

**FIGURE 8:**
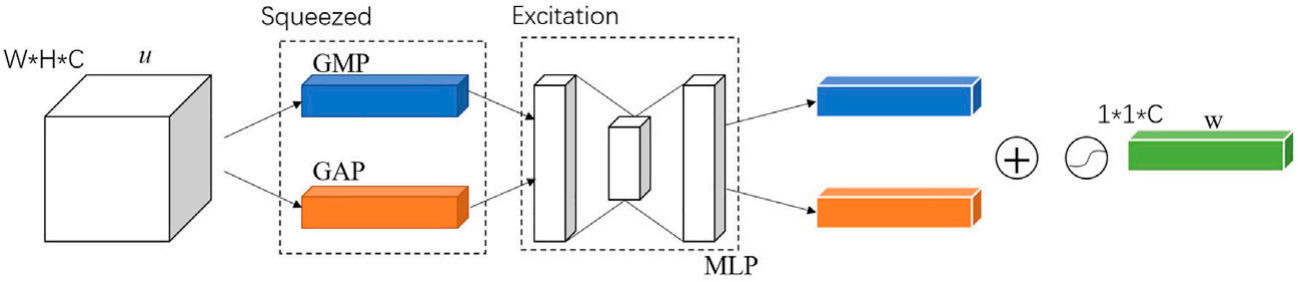
Channel attention module.

The channel attention mechanism, which is used to construct correlations between channels by performing dimensionality reduction in channel dimensions, enables weighting of critical channels. The channel attention mechanism is a simple and effective module that can be embedded in any part of the network. It can reduce the redundancy of channels, enhance channels that are important to the task and weakening channel dimensions that have less impact on the task. The channel attention module is shown in Figure 8.

The input to the channel attention module is the features *u*, which is a feature vector with channel dimension *C*, length *H* and width *W*. First, *u* is squeezing in the spatial dimension, keeping the channel dimension *C*, and squeezing the spatial dimensions *W* and *H* to *1* and *1*, respectively, so as to obtain a *1* × *1* × *C* vector, which is called the squeezing part. Then, the squeezed vector is fed into the Multiple Perception Machine (MLP) to recover the channel dimension and output a *1 × 1 × C* vector, which is called excitation. Finally, outputs of excitation are fused by elementwise addition. The fusion result is activated with a nonlinear activation function to obtain a *1 × 1 × C* channel attention vector ***w***.

The squeeze process is implemented by global pooling. The squeeze process is actually a squeeze of the spatial domain, reducing the effect of spatial location on the channel dimension, and obtaining the complete information of the channel domain. Both Global Average Pooling (GAP) and Global Max Pooling (GMP) are used, shown in Figure 8. The excitation is an Auto Encoder using MLP to extract features from important channels in the channel domain. The Auto Encoder is implemented using a 1 × 1 convolution kernel. In the Auto Encoder, the dimensions of the input and output layers are the same, and the dimension of the intermediate hidden layers must be smaller than that of the input and output layers to achieve dimensionality reduction and eliminate redundant channels in the input layer.

In translation and rotation, the response of elements near diagonal is stronger. Therefore, elements near diagonal contain more information. The special spatial structure of the diagonal has an important influence on the final result of pose estimation. We use a spatial attention mechanism to weight the elements at different locations in space to strengthen the influence of diagonal elements on pose estimation and weaken the influence of non-diagonal elements.

**FIGURE 9:**
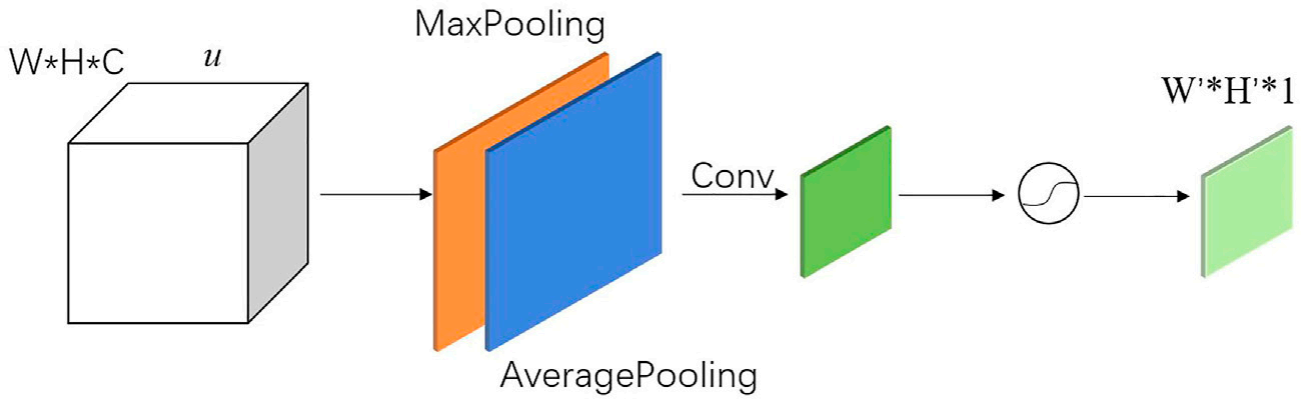
Spatial attention.

**FIGURE 10:**
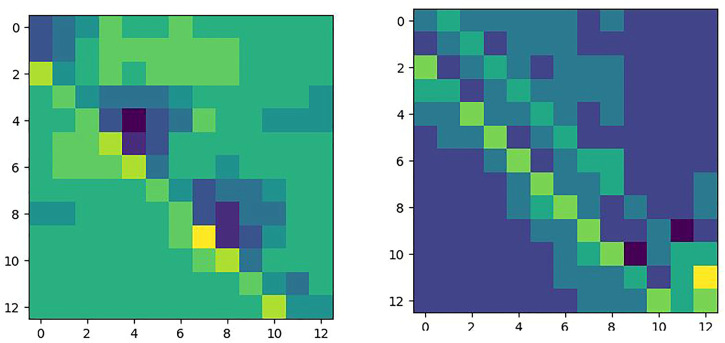
Spatial attention in translation and rotation movement.

The implementation of the spatial attention mechanism is shown in Figure 9. Two *W × H × 1* features with the same spatial resolution as the input *u* are generated by using maximum pooling and average pooling in dimensions, respectively. Subsequently, features are concatenated in the dimension to obtain a *W × H × 2* feature. Next, the new feature is convolved to reduce its channel dimension to 1 dimension. Finally, the results are activated by the activation function, and a *W' × H' × 1* feature is output as weights, which can be directly used in output of the pose calculate network $u^{\prime }$ . The results of spatial attention are shown in Figure 10.

The output of spatial attention is not directly applied to the input *u*, but to the pose estimation $u^{\prime }$ , so that the input includes both the initial *u* and the pose estimation $u^{\prime }$ . The *average* and *mean* results of average pooling are fused by channel dimension concatenation, followed by convolution and activation function to obtain the weights *w*, which are finally weighted by the pose estimation $u^{\prime }$ .

## 4 Experiment

The hardware consists of Xeon E5 CPU, Nvidia GeForce RTX 2080Ti GPU and 32G of memory capacity. The software platform uses Windows, programming using Python 3.8.6, deep learning framework by Google’s open source framework Tensorflow 2.2.0 and Keras.

Sequences of colour video images from visual odometry under the Open Data KITTI are used as experimental data, with an image size of 1280 *×* 640 and a total of 11 sequences. Each sequence ranges from 500 to 5,000 m in length. All inputs are used as image pairs for the time interval *i* is 1. The datasets with different sequences are randomly sampled for the input data to ensure a uniform distribution of the training. The FPN PoseEstimateNet was trained with batch size of 4 and a learning rate of 0.0001, using the 00 sequence as the training set, which consists of 512 image pairs randomly sampled from the 00 sequence for 1 epoch.

The method consists of two different stages: the feature extractor and the pose calculate network. The method has two different types of labels: similarity label and pose label. The similarity labels are coded using 0 and 1, 0 for similarity and 1 for dissimilarity. In the experiments, image pairs with time interval *i* < 5 are positive samples and those with time interval *i* > 10 are negative samples. The ratio of positive to negative sample is 1:1. As all the pose labels provided in the KITTI dataset are a coordinate matrix with the coordinates of frame 0 as the origin, the pose represented is the absolute pose at the origin of the coordinates of frame 0. The predicted pose is a relative pose between two frames. So the original labels provided by the KITTI dataset need to be transformed from absolute to relative pose. 3 degrees of freedom (DoF) of the vehicle are used for the pose labels.

If the absolute pose of the *n*th frame is represented as ***T***_***n***_, the absolute pose matrix of the *n*th *+* 1 frame is represented as ***T***_***n+1***_, and the relative pose of the 2 frames is represented as ***T***_***r***_, the relative pose transformation is shown in Eq. 7. The ***T***_***r***_ is constructed by the rotation matrix $R=\left[\begin{array}{ccc} R_{\text{r}1} & R_{\text{r}2} & R_{\text{r}3}\\ R_{\text{r}4} & R_{\text{r}5} & R_{\text{r}6}\\ R_{\text{r}7} & R_{\text{r}8} & R_{\text{r}9} \end{array}\right]$ and the translation matrix $T=\left[\begin{array}{c} x_{\text{r}}\\ y_{\text{r}}\\ z_{\text{r}} \end{array}\right]$ .

$$T_{\text{r}}=T_{\text{n}}^{-1}T_{\text{n}+1}$$ (7)

$$T_{r}=\left[\begin{array}{cc} R & T\\ 0 & 1 \end{array}\right]$$

During the pose calculation, the rotation matrix ***R*** can be used to represent the rotation around different axes, and the translation matrix ***T*** can be used to represent the translation along different directions. However, there are some redundancy in the rotation matrix ***R*** and the orthogonality constraint is difficult to realize in deep learning. The rotation needs to be convert into Eulerian angle suitable for deep learning. The conversion of rotation matrix ***R*** is shown in Eqs 8, 9, 10.

$$\alpha =\text{arctan}\left(-R_{\text{r}7}/\sqrt{R_{r1}^{2}+R_{r4}^{2}}\right)$$ (8)

$$\beta =\text{arctan}\left(R_{\text{r}4}/R_{\text{r}1}\right)$$ (9)

$$\gamma =\text{arctan}\left(R_{\text{r}8}/R_{\text{r}9}\right)$$ (10)

The KITTI dataset uses the right hand coordinate system, and the vehicle movement direction is *z axis*. In most cases, the translation in *z axis* is much larger than that in the other directions. Similarly, the rotation in the *zoy* plane will be much larger than that in the other planes.

**FIGURE 11:**
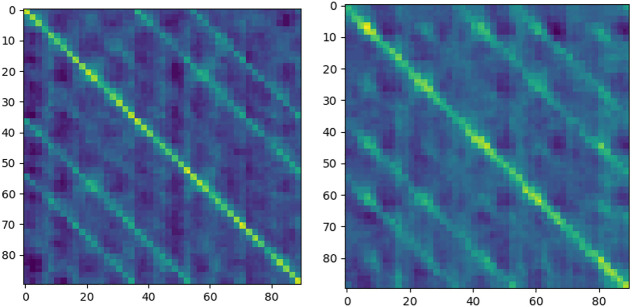
Correlation matrix *φ* in translation (left) and rotation (right).

The correlation matrix *φ* for FPN PoseEstimateNet is shown in Figure 11. It is close to a diagonal matrix. The elements on the diagonal in the translation and rotation movement have essentially trend.

**FIGURE 12:**
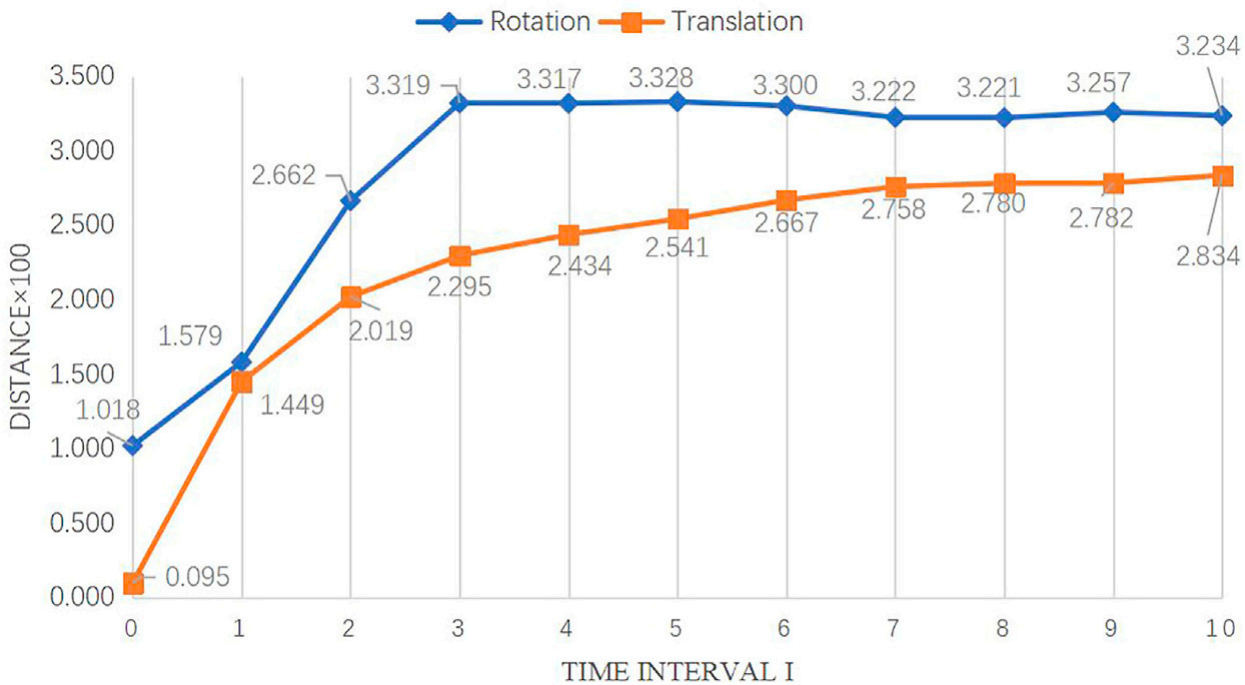
Trend of distance over time interval.

The difference in distance of rotation and translation for different time intervals is shown in Figure 12. As the time interval increases, the difference of rotation and translation increases, indicating that the feature extractor is able to perform similarity analysis. At the same time, the *distance* under rotation variation increases significantly in a short period of time, and then tends to stability; the *distance* under translation variation increases significantly in a shorter period of time, and then still increases slowly and stable off gradually. Figure 12 shows that the method is good at identifying differences for translation changes in any length of time, while it is good at identifying differences for rotation changes in shorter periods of time, but poor at identifying differences for longer periods of time.

**FIGURE 13:**
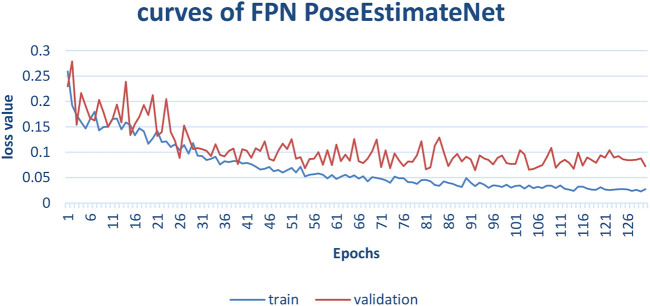
FPN PoseEstimateNet train and valid curves.

The training process is shown in Figure 13. In the early 50 epochs, the validation curve is close to the training curve. Then, the validation loss is larger than the training loss. The training loss decreases rapidly and converges gradually. Due to the variation of different sequences and the difference of vehicle speed, there is an obvious jitter in the validation curve.

Because the FPN PoseEstimateNet lacks a back-end optimisation, the method is used for short distance for pose estimation. Absolute Pose Error (APE) is used as the evaluation criterion. The APE includes Absolute Transpose Error (ATE) and Absolute Rotate Error (ARE). The smaller the error, the closer the predicted pose is to the truth. The data used in the experiments are 200 consecutive images.

The results show that the prediction results are quite different for different sequences, shown in Table 3. On the one hand, this is because only the 00 sequence is used as the training set, so it has certain limitations in generalization. On the other hand, the performance is limited by the network size.

**TABLE 3:** Prediction accuracy of different series.

Serials	FPN PoseEstimateNet		FlowNet	
	ATE (m)	ARE (°)	ATE(m)	ARE (°)
00	9.41	4.87	5.43	4.62
03	12.07	1.38	18.05	8.82
05	8.96	4.26	7.92	3.54
07	21.55	5.92	23.61	4.11

As shown in Table 4, FlowNet uses a fully connected layer with weight of 581M, which can improve the accuracy and reduce the inference speed. The parameter of FPN PoseEstimateNet is only 101.6M, but it can accelerate the inference.

**TABLE 4:** Inference speed and parameters for different networks.

	FPN PoseEstimateNet	FlowNet
Inference speed (FPS)	6	2
Parameter size (M)	101.6	581

**FIGURE 14:**
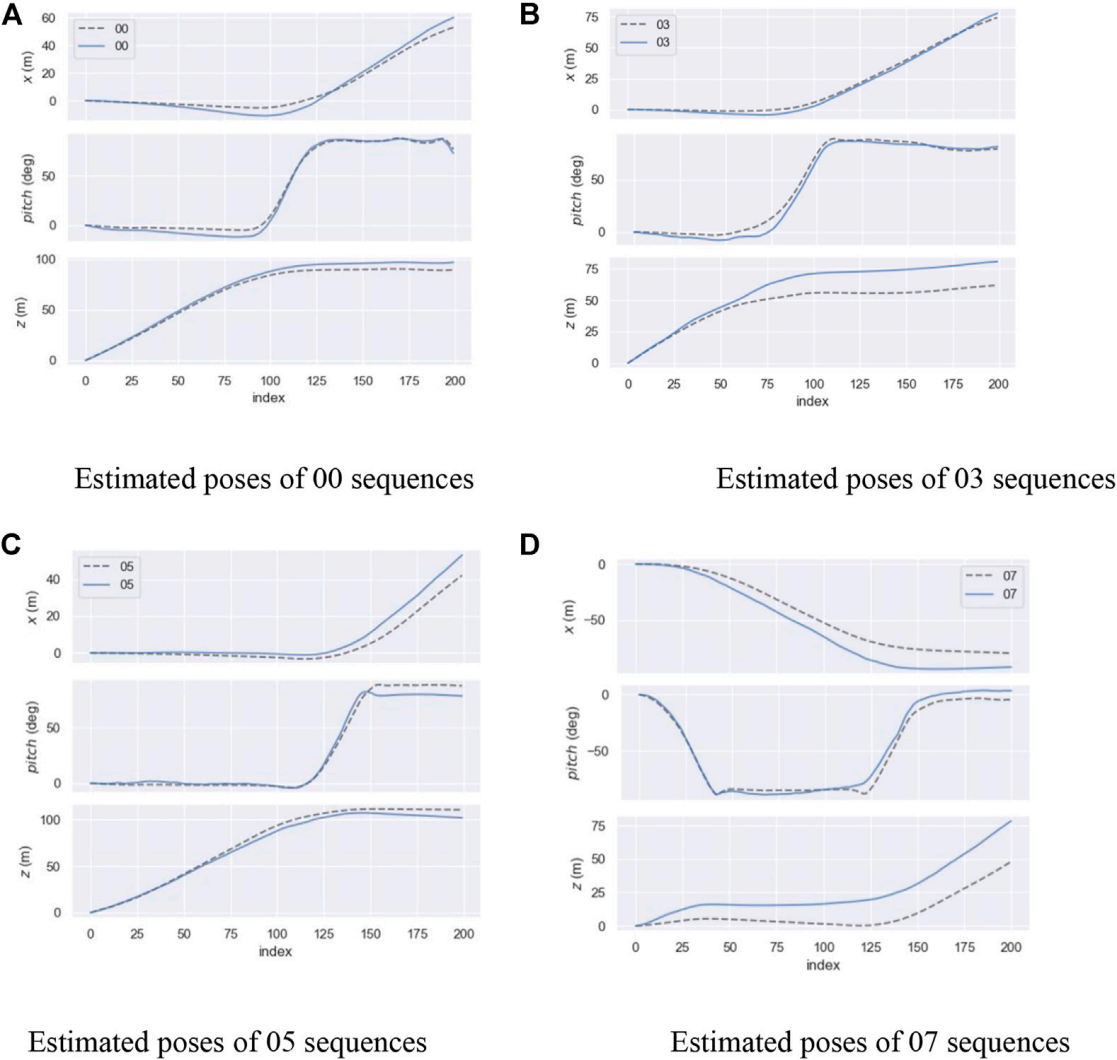
Results of estimated pose. **(A)** Estimated poses of 00 sequences. **(B)** Estimated poses of 03 sequences. **(C)** Estimated poses of 05 sequences. **(D)** Estimated poses of 07 sequences.

Figure 14 shows the performance of the FPN PoseEstimateNet on different sequences. Because the pose transformation is in *xoz* plane during movement, Figure 14 shows only the estimated curves of translation and rotation in *xoz* plane. The top is the translation in the *x* axis, the middle is the rotation, and the bottom is the translation in the *z* axis. The solid line is the prediction and the dashed line is the truth. It can be seen that FPN PoseEstimateNet successfully predicts the trend of pose changes in all sequences. However, when the rotation was started and stopped, there will be errors in the predicted rotation. In these moments, the translation error will also increase, and the translation is usually larger than the truth. The FPN PoseEstimateNet has a small error for fast rotations in a short time.

## 5 Conclusion

We propose a novel approach for vehicle pose estimation using a monocular camera. This method combines deep learning with traditional pose estimation algorithms through the Siamese network. The network runs at a speed of 6 FPS, and the parameter size is 101.6 M. In different sequences, the angle error is within 8.26° and the maximum translation error is within 31.55 m. However, the error in predicted pose will accumulate and has a significant impact on the long-term pose estimation. In order to optimize the global trajectory, a correction process must be adopted. On the other hand, the pose estimation of the vehicle is 3 DoF, which includes translation and rotation in the driving plane, while the translation and rotation in other directions are ignored. However, for the movement of unmanned aerial vehicles (UAVs) and other objects, pose estimation of 6 DoF is needed.

## Data Availability

Publicly available datasets were analyzed in this study. This data can be found here: http://www.cvlibs.net/datasets/kitti/raw_data.php.
